# Association between antibiotic use and the onset of giant cell arteritis and polymyalgia rheumatica: A nested case–control study from E3N‐European Prospective Investigation into Cancer and Nutrition

**DOI:** 10.1111/joim.70000

**Published:** 2025-09-02

**Authors:** Lucas Pacoureau, François Barde, Amandine Gelot, Alexis Elbaz, Agnès Fournier, Yann Nguyen, Raphaèle Seror

**Affiliations:** ^1^ Université Paris‐Saclay, UVSQ, Inserm CESP U1018, Gustave Roussy Villejuif France; ^2^ Service de Médecine Interne, Hôpital Bicêtre, AP‐HP.Sud, Université Paris‐Saclay Le Kremlin‐Bicêtre France; ^3^ Service de Médecine Interne, Hôpital Beaujon, AP‐HP.Nord, Université Paris Cité Paris France; ^4^ Centre de Recherche en Epidémiologie et Statistiques (CRESS) Unité Inserm 1153, Université de Paris Cité Paris France; ^5^ Department of Rheumatology Hôpital Bicêtre, AP‐HP.Sud Le Kremlin‐Bicêtre France

**Keywords:** antibiotic, disease risk factor, E3N, giant cell arteritis, infections, nested case–control study, polymyalgia rheumatica, trigger

## Abstract

**Objectives:**

To assess the association between infections, assessed by antibiotic reimbursement, and the occurrence of giant cell arteritis (GCA) and/or polymyalgia rheumatica (PMR).

**Methods:**

We conducted a nested case–control study from the French cohort E3N‐European Prospective Investigation into Cancer and Nutrition, which has followed 98,995 women since 1990. Cases, defined as patients who developed GCA and/or PMR during follow‐up, were matched with 20 controls on age and vital status. Infections prior to index date, defined by ≥1 antibiotic reimbursement on the medication claims reimbursement database, were compared between groups using conditional logistic regression models, adjusted for potential confounders. Different time periods before the index date and different antibiotic classes were compared.

**Results:**

A total of 428 GCA/PMR cases (113 GCA, 232 PMR, 83 undefined) were compared to 8560 matched controls. Compared to controls, GCA/PMR cases had higher odds to have any infection in the [0–24] months prior to index date (aOR [95% CI] 1.22 [1.00–1.51]). Considering the 6‐month periods prior to index date, the association was stronger when close to index date (1.18 [0.94–1.47]; 0.95 [0.75–1.19] for [0–6] and [18–24] months, respectively). This association was only found among GCA cases (1.63 [1.08–2.48] for [0–24] months), but not among PMR cases. Quinolone reimbursements were the most associated with subsequent GCA (2.07 [1.23–3.49] for [0–12] months).

**Conclusion:**

Compared to controls, GCA patients were at higher risk of having used antibiotics in the 24 months prior to the diagnosis. Infections or a disbalanced microbiome could act as a trigger of the disease, although a reverse causation bias cannot be excluded.

## Graphical abstract

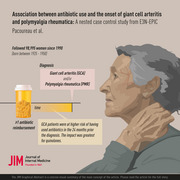

## Introduction

Giant cell arteritis (GCA) is the most frequent large‐vessel vasculitis, affecting mostly individuals over the age of 50 years [1, 2, 3]. Its frequent association with polymyalgia rheumatica (PMR) suggests a common pathophysiological mechanism.

Etiological mechanisms of those diseases remain unclear and are discussed. Several elements suggest a link with environmental factors, as epidemiological studies in different geographical areas [1, 4, 5] have observed epidemic peaks and seasonal variations in the incidence of GCA or PMR. In addition, GCA's pathogenesis would involve vascular parietal inflammation in response to a hypothetic antigenic trigger not yet identified [6, 7], driving a Th1 and Th17 lymphocyte adaptive immune response [8], two classical pathways of anti‐infectious response.

Therefore, the hypothesis of an infectious trigger of these diseases in relation to a predisposed genetic background (HLA DR4) [9] has emerged.

Several infectious agents have thus been suspected, among them varicella zoster virus [10, 11], *Mycoplasma pneumoniae*, *Chlamydia pneumoniae*, or parvovirus B19 [12, 13, 14]. However, data remained inconsistent [15, 16, 17], as most studies have focused on the direct search for a pathogen on temporal artery biopsy sections, with no standardized methods to confirm the presence of the infectious agent (different PCR expansion targets, different expansion thresholds), which may in part explain the absence of an unequivocal response.

Epidemiological studies (retrospective cohort or case–control study), mostly relying on coding data, both for cases and infection definitions [18, 19, 20, 21], aimed to assess this association. However, the definition and the timing of the exposition differed. Onset of GCA tended to be associated with prior overall infection, with a higher risk in cases of infections close to the diagnosis date [18, 19, 22]. Same results were found by Brault et al. [21] about community‐ and hospital‐treated infections, with a higher risk of GCA when infections occurred during the year before diagnosis. None of this study focused on antibiotic class used.

Through a nested case–control study of a large prospective cohort, we aimed to assess the association between infections, defined by use of antibiotics, and the onset of GCA/PMR. We also aimed to determine whether a temporal link can be determined by analyzing different exposure windows and whether the risk could differ according to the antibiotic class.

## Materials and methods

### Study population

The E3N cohort study (*Etude Epidémiologique auprès des femmes de la Mutuelle générale de l'Education Nationale*, MGEN) is a large French prospective cohort study designed to investigate the link between lifestyle, diet, and environmental factors and chronic diseases such as cancer or diabetes [23]. It is the French component of the European Prospective Investigation into Cancer and Nutrition.

It includes 98,995 healthy French women born between 1925 and 1950 affiliated with a national health insurance plan for workers in the French education system [23]. Participants were recruited in 1990 and completed mailed questionnaires every 2 years since (up to date 13 questionnaires, last in 2021) to update their health‐related information, lifestyle characteristics, diet, and newly diagnosed diseases. Since 2004, linkage with a drug reimbursement claims database has been available from MGEN. The average response rate per follow‐up questionnaire is 83%, and the total proportion of patients lost to follow‐up since 1990 is <3%.

### Case definition

The identification of GCA/PMR cases is described elsewhere [24]. Briefly, women who self‐reported a diagnosis of GCA and/or PMR (with no distinction between diseases) in three follow‐up questionnaires (Q9, Q10, and Q11, respectively, in 2008, 2011, and 2014) were sent a specific validation questionnaire in 2019, with a reminder in 2020 (potential cases *N* = 1143, response rate = 71.7%).

Women were considered cases in two situations. First, if they confirmed having GCA/PMR in the specific validation questionnaire and fulfilled all the following criteria: (1) GCA/PMR diagnosis was confirmed by a physician (general practitioner and/or specialist physician), (2) they were aged 50 years or older at time of diagnosis, and (3) they had been treated with glucocorticoids (GC) for over 3 months (self‐reported or confirmed by drug reimbursements for diagnosis after 2004). Other prospective cohorts have used these criteria to identify GCA and/or PMR patients [25]. This method of validation provided satisfying accuracy to detect cases (agreement of 89.6% with medical chart review for GCA and/or PMR classification). In addition, potential cases who did not answer the specific validation questionnaire but who self‐declared having GCA and/or PMR at the three follow‐up questionnaires were considered cases if they were aged ≥50 years at the age of diagnosis and received at least 3 months of GC, according to the drug claims database. This validation method also enabled cases to be detected with satisfactory accuracy (agreement of 92.8% with medical chart review for GCA/PMR classification). A similar method has been used to identify rheumatoid arthritis cases in the E3N cohort [26, 27]. Thus, all cases were validated by one of the two methods.

The index date was defined as the self‐reported date of GCA/PMR diagnosis in the validation questionnaire or in the three follow‐up questionnaires (for women who did not answer the validation questionnaire). In order to include a 2‐year period of exposure for all cases, those with an index date before January 1, 2006, were excluded, as the linkage with the reimbursement database began in January 2004.

### Controls

For each case, 20 controls, who never self‐declared any diagnosis of GCA and/or PMR, were matched on year of birth (±1 year) and vital status at the time of the specific questionnaire (2019), using a SAS macro [28]. As the self‐report of GCA and/or PMR only occurred during Q9, Q10, and Q11, we excluded women who did not answer any of those three questionnaires. For controls, the index date was the one of their respective matched cases.

### Infection assessment

Exposure was defined as any episode of infection, defined by any antibiotic reimbursement prior to the index date. Antibiotic drugs included were the most commonly prescribed and were defined according to the ATC classification (Appendix S1). Data were obtained from the drug reimbursement claim database (MGEN), which contains complete information on drug reimbursements since January 1, 2004. Women who did not receive any reimbursement after 2019 were excluded from our analyses, as they may no longer be covered by the MGEN insurance, and their exposure could no longer be assessed.

Two additional exposure variables were considered: the number of antibiotic reimbursements and the most frequently used specific antibiotic classes (i.e., beta‐lactams, quinolones, and macrolides defined according to the ATC classification).

### Covariates

Data on education level (<high school, up to two‐level university, or two‐level university or more) and socio‐professional category (teacher, higher managerial and professional occupations, intermediate occupation, unemployed, and other) were assessed at inclusion in the cohort.

Smoking status (past, current, and never) was recorded and updated at each questionnaire. Self‐reported height and weight were used to calculate body mass index (BMI) at each questionnaire, defined as weight (kg) divided by squared height (m^2^). BMI was divided into four categories (<18.5, [18.5–25], [25–30], ≥30 kg/m^2^). Type 2 diabetes was based on self‐reports, then validated through a specific questionnaire; cases occurring after 2004 were identified using the MGEN reimbursement database [29].

History of cancer was identified based on three different sources: self‐reported cancer history in follow‐up questionnaires, spontaneous declaration by relatives, and causes of death from a national registry (available until 2014).

For the following time‐varying variables (smoking status, Type 2 diabetes, history of cancer, and BMI), we used the closest value to 2 years before the index date so that confounders were assessed before the exposure.

### Statistical analysis

Baseline characteristics (i.e., at cohort inclusion for educational level and socio‐professional category and 2 years before index date for other variables) are described as means (standard deviation [SD]) for continuous variables and *n* (%) for categorical variables.

Conditional logistic regression models with and without adjustment on educational level, socio‐professional category, BMI, smoking status, and history of Type 2 diabetes and cancer were used to determine if infections, defined by antibiotic reimbursements, were associated with the risk of incident GCA/PMR. The matched‐group identifier was specified as the clustering variable in the conditional logistic regressions to account for the matched case–control design. Different time periods were considered prior to the index date: by 6 months ([0–6], [6–12], [12–18], [18–24] months), by 12 months ([0–12], [12–24] months), or [0–24] months. As antibiotic reimbursements for each period were not independent for models by 6 or 12 months, all periods were included in the same model. Odds ratios (ORs) and 95% confidence intervals (95% CIs) were estimated for each period.

The same models were used to evaluate the association between the most frequently used antibiotic classes (beta‐lactams, quinolones, macrolides, and related; see Appendix S1) and incident GCA/PMR during the same time periods.

To examine a possible dose–response relationship between the number of previous infections and incident GCA/PMR, we used a separate logistic regression model to estimate ORs (95% CI) associated with the number of antibiotic reimbursements (0, 1, and >1 reimbursement, respectively) prior to the index date, by 12‐month periods.

To analyze separately the risk of GCA (with or without signs of PMR) and PMR (without signs of GCA), we used the same adjusted models, assessing GCA and PMR separately, in which GCA and PMR cases were compared to their respective matched controls.

To avoid reverse causation bias for cases in whom GC was prescribed before diagnosis, we performed a sensitivity analysis, in which a revised index date for these cases was defined as the start date of a >3‐month course of GC instead of the self‐reported diagnosis date. GC reimbursement data were available in the drug claims database, and we identified GC reimbursements over a 1‐year period around the date of diagnosis. The start date of the >3‐month course of GC was defined by the earliest date of GC reimbursement, followed by at least one GC reimbursement within 3 months.

Missing data were imputed only for confounding factors, if they occurred in less than 5% of subjects, using data from the previous questionnaire for BMI and smoking status or the modal category for education level. Otherwise, when missing data occurred in more than 5% of subjects, a separate “missing category” was created for the variable, without imputation. This is the usual and validated procedure used by E3N teams [30, 31].

Analyses were carried out using the Statistical Analysis Systems software package, version 9.4 (SAS Institute Inc.).

## Results

### Study population

**Fig. 1:**
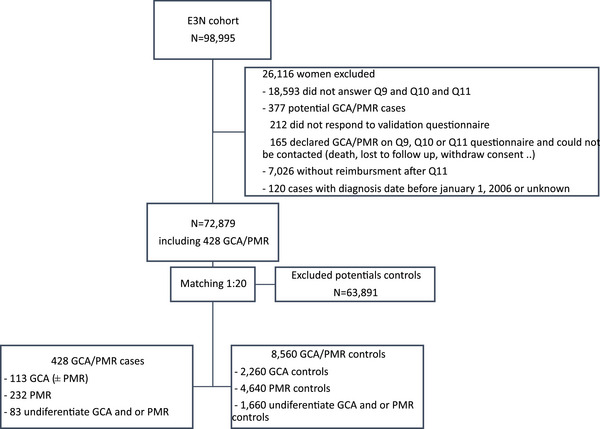
Flow chart. GCA, giant cell arteritis; PMR, polymyalgia rheumatica.

We identified 428 cases of GCA and/or PMR diagnosed after January 1, 2006. Among them, 113 (26.4%) were identified as GCA cases, 232 (54.2%) as PMR cases, and the remaining 83 (19.4%) cases could not be differentiated (GCA and/or PMR). Each of them was matched with 20 controls, resulting in a total population of 8988 patients (Fig. 1).

The main characteristics of the study population according to GCA/PMR status at index date are summarized in Table 1.

**Table 1:** Characteristics of GCA/PMR cases and controls at index date.

	Matched controls^a^ (*N* = 8560)	GCA/PMR cases (*N* = 428)	GCA cases (*N* = 113)	PMR cases (*N* = 232)
**Age at index date (years)**	72.1 (6.1)	72.1 (6.1)	72.9 (6.1)	70.8 (5.8)
**Education level (%)**				
High school	984 (11.5)	44 (10.3)	10 (8.9)	22 (9.5)
Up to 2‐level university	4382 (51.2)	229 (53.5)	51 (45.1)	128 (55.2)
≥3 level university	2866 (33.5)	142 (33.2)	46 (40.7)	78 (33.6)
Not available	328 (3.8)	13 (3.0)	6 (5.3)	4 (1.7)
**Socio‐professional category (%)**				
Teacher	5870 (68.6)	291 (68.0)	75 (66.4)	159 (68.5)
Higher managerial and professional occupations	219 (2.6)	17 (4.0)	4 (3.6)	7 (3.0)
Intermediate occupation	1297 (15.2)	62 (14.5)	18 (15.9)	38 (16.4)
Unemployed	169 (2.0)	12 (2.8)	4 (3.6)	4 (1.7)
Other	47 (0.4)	6 (1.4)	1 (0.9)	3 (1.3)
Not available	958 (11.2)	40 (9.3)	11 (9.7)	21 (9.1)
**Body mass index (kg/m^2^)**				
<18.5	302 (3.5)	12 (2.8)	4 (3.6)	6 (2.6)
[18.5–25]	5457 (63.8)	282 (65.9)	86 (76.1)	147 (63.4)
[25–30]	2189 (25.6)	104 (24.3)	18 (15.9)	60 (25.9)
≥30	608 (7.1)	30 (7.0)	5 (4.4)	19 (8.2)
Not available	4 (0.0)	0 (0.0)	0 (0.0)	0 (0.0)
**Smoking status (%)**				
Non smoker	4832 (56.4)	234 (54.7)	56 (49.6)	134 (57.8)
Former smoker	3122 (36.5)	156 (36.4)	48 (42.5)	75 (32.3)
Current smoker	604 (7.1)	38 (8.9)	9 (8.0)	23 (9.9)
Not available	2 (0.0)	0 (0.0)	0 (0.0)	0 (0.0)
**Type 2 diabetes (%)**	420 (4.9)	16 (3.7)	4 (3.5)	9 (3.9)
**History of cancer (%) (*N* = 8970)**	1518 (17.7)	60 (14.0)	19 (16.8)	30 (12.9)
**≥1 antibiotic reimbursement in the year prior to index date (%)**				
All	3637 (42.5)	199 (46.5)	59 (52.2)	108 (46.6)
Beta‐lactam	2345 (27.4)	131 (30.6)	43 (38.1)	70 (30.2)
Quinolone	880 (10.3)	45 (10.5)	21 (18.6)	25 (10.8)
Macrolide	997 (11.6)	57 (13.3)	18 (15.9)	26 (11.2)

*Note N*: (%) for categorical variables. Mean (SD) for continuous variables.

Abbreviations: BMI, body mass index; GCA, giant cell arteritis; PMR, polymyalgia rheumatica

^a^ Controls were matched on year of birth (±1 year).

For cases and controls, mean (±SD) age at GCA/PMR diagnosis or index date was 72.1 (6.1) years. No differences were observed between GCA/PMR cases and controls regarding their education level nor their socio‐professional category. More than half of GCA/PMR cases and controls were non‐smokers at the time of diagnosis or index date (54.7% and 56.4%, respectively). GCA/PMR cases were less likely to have a history of cancer (14.0% vs. 17.7%, respectively) or Type 2 diabetes (3.7% vs. 4.9%, respectively). During the year before the diagnosis date of GCA/PMR, 46.5%, 30.6%, and 10.5% received, respectively, antibiotics, beta‐lactam, and quinolone, whereas 42.5%, 27.4%, and 10.3% of controls received, respectively, antibiotics, beta‐lactam, and quinolone, in the year before index date.

### Overall antibiotic use and the risk of GCA/PMR

**Fig. 2:**
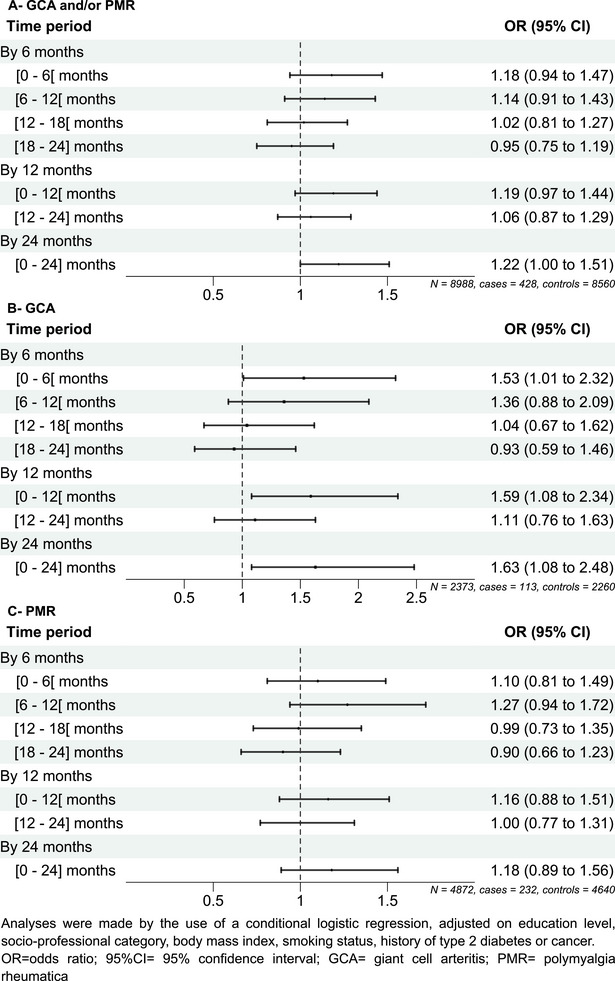
Association between any antibiotic reimbursement and incident (A) GCA and/or PMR (B), GCA, and (C) PMR alone by period. 95% CI, 95% confidence interval; GCA, giant cell arteritis; PMR, polymyalgia rheumatica.

The associations between any antibiotic use and GCA/PMR according to different periods are shown in Table 2 and Fig. 2. Considering the [0–24] month period prior to the index date, antibiotic use was associated with a higher risk of GCA/PMR in multivariate models (aOR [95% CI] 1.22 [1.00–1.51]). Associations were stronger, both in uni‐ and multivariate models, for the [0–6] (aOR [95% CI] 1.18 [0.94–1.47]) and [0–12] (aOR [95% CI] 1.19 [0.97–1.44]) month periods prior to the index date compared to other periods, and ORs tended to decrease as the delay between the exposure and the index date increased.

**Table 2:** Odds ratios (95% confidence intervals) between antibiotics consumption by period and incident cases of GCA/PMR, GCA, or PMR alone.

	Overall				GCA				PMR			
Time period prior to index date	Cases *N* = 428	Controls *N* = 8560	Univariate analysis OR (95% CI)	Multivariate analysis^a^ OR (95% CI)	Cases *N* = 113	Controls *N* = 2260	Univariate analysis OR (95% CI)	Multivariate analysis^a^ OR (95% CI)	Cases *N* = 232	Controls *N* = 4640	Univariate analysis OR (95% CI)	Multivariate analysis^a^ OR (95% CI)
**By 6 months**												
[0–6] months	132 (30.8)	2334 (27.3)	1.19 (0.96–1.47)	1.18 (0.94–1.47)	42 (38.2)	627 (27.7)	1.55 (1.04–2.31)	**1.53 (1.01–2.32)**	69 (29.7)	1278 (27.5)	1.12 (0.83–1.49)	1.10 (0.81–1.49)
[6–12] months	126 (29.4)	2265 (26.5)	1.16 (0.94–1.44)	1.14 (0.91–1.43)	36 (31.9)	555 (24.6)	1.43 (0.96–2.15)	1.36 (0.88–2.09)	74 (31.9)	1262 (27.2)	1.26 (0.95–1.68)	1.27 (0.94–1.72)
[12–18] months	121 (28.3)	2338 (27.3)	1.05 (0.85–1.30)	1.02 (0.81–1.27)	33 (29.2)	600 (26.6)	1.14 (0.75–1.73)	1.04 (0.67–1.62)	66 (28.5)	1287 (27.7)	1.04 (0.77–1.39)	0.99 (0.73–1.35)
[18–24] months	111 (25.9)	2294 (26.8)	0.99 (0.80–1.24)	0.95 (0.75–1.19)	30 (26.6)	587 (26.0)	1.03 (0.67–1.58)	0.93 (0.59–1.46)	62 (26.7)	1290 (27.8)	0.95 (0.70–1.28)	0.90 (0.66–1.23)
**By 12 months**												
[0–12] months	199 (46.5)	3637 (42.5)	1.18 (0.97–1.43)	1.19 (0.97–1.44)	59 (52.2)	945 (41.8)	1.53 (1.04–2.23)	**1.59 (1.08–2.34)**	108 (46.6)	2006 (43.2)	1.15 (0.88–1.50)	1.16 (0.88–1.51)
[12–24] months	188 (43.9)	3651 (42.7)	1.05 (0.87–1.28)	1.06 (0.87–1.29)	49 (43.4)	937 (41.5)	1.08 (0.74–1.58)	1.11 (0.76–1.63)	101 (43.5)	2024 (43.6)	1.00 (0.76–1.30)	1.00 (0.77–1.31)
**By 24 months**												
[0–24] months	281 (65.7)	5242 (61.2)	1.21 (0.99–1.49)	**1.22 (1.00–1.51)**	80 (70.8)	1366 (60.4)	1.59 (1.05–2.41)	**1.63 (1.08–2.48)**	152 (65.5)	2876 (62.0)	1.17 (0.88–1.55)	1.18 (0.89–1.56)

*Note*: Significant values are indicated in bold.

Abbreviations: 95% CI, 95% confidence interval; GCA, giant cell arteritis; OR, odds ratio; PMR, polymyalgia rheumatica.

^a^ Analyses adjusted for educational level, socio‐professional category, body mass index, smoking status, Type 2 diabetes, and history of cancer.

In analyses restricted to PMR cases (*n* = 232), there was no association between antibiotic use in uni‐ or multivariate analyses, regardless of the period (Table 2, Fig. 2). In analyses restricted to GCA cases (*n* = 113), antibiotic use over the [0–24] month period was significantly associated with GCA (aOR [95% CI] 1.63 [1.08–2.48]); this association was stronger and significant for the [0–6] (aOR [95% CI] 1.53 [1.01–2.32]) and [0–12] (aOR [95% CI] 1.59 [1.08–2.34]) month periods.

**Fig. 3:**
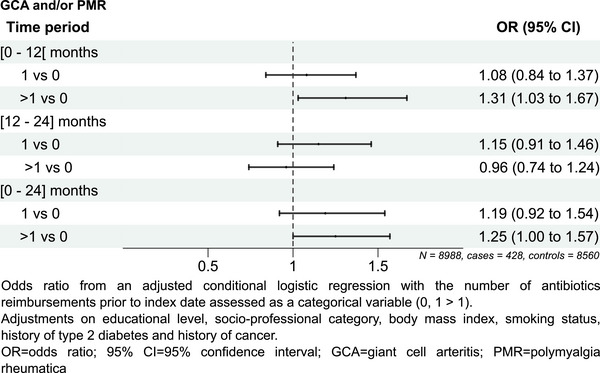
Association between the number of antibiotics intakes prior to diagnosis or index date and GCA/PMR by period. 95% CI, 95% confidence interval; GCA, giant cell arteritis; PMR, polymyalgia rheumatica.

Associations between the number of antibiotic reimbursements and risk of GCA/PMR according to the different periods are shown in Fig. 3. Patients with more than one antibiotic reimbursement had a significantly higher risk of developing GCA/PMR than patients with no antibiotic reimbursement in the [0–12] (aOR [95% CI] 1.31 [1.03–1.67]) and [0–24] (aOR [95% CI] 1.25 [1.00–1.57]) month periods. No significant association was observed for exposures over the [12–24] month period or for 1 reimbursement.

### Class of antibiotics and risk of GCA/PMR

Associations between beta‐lactam, quinolone, and macrolide reimbursements and risk of GCA and/or PMR are presented in Table 3. We found a positive and significant association for beta‐lactam prescriptions in the [0–24] month period (aOR [95% CI] 1.25 [1.03–1.52]).

**Table 3:** Association between beta‐lactam, quinolone, and macrolide consumption by period and incident GCA/PMR, GCA, or PMR alone.

	Beta‐lactam			Quinolone			Macrolide		
Time period prior to index date	Cases	Controls	aOR^a^ (95% CI)	Cases	Controls	aOR^a^ (95% CI)	Cases	Controls	aOR^a^_ (95% CI)
**Overall (cases** = **428 controls** = **8560)**									
[0–12] months	131 (30.6)	2345 (27.4)	1.16 (0.94–1.44)	45 (10.5)	880 (10.3)	1.08 (0.78–1.50)	57 (13.3)	997 (11.6)	1.17 (0.88–1.57)
[12–24] months	128 (29.9)	2380 (27.8)	1.08 (0.87–1.35)	35 (8.2)	832 (9.7)	0.82 (0.57–1.18)	51 (11.9)	1011 (11.8)	0.99 (0.73–1.35)
[0–24] months	209 (48.8)	3728 (43.6)	**1.25 (1.03–1.52)**	69 (16.1)	1430 (16.7)	0.96 (0.74–1.25)	95 (22.2)	1,756 (20.5)	1.11 (0.88–1.41)
**GCA (cases** = **113, controls** = **2260)**									
[0–12] months	43 (38.1)	617 (27.3)	**1.73 (1.15–2.61)**	21 (18.6)	230 (10.2)	**2.07 (1.23–3.49)**	18 (15.9)	243 (10.8)	1.70 (0.99–2.90)
[12–24] months	32 (28.3)	622 (27.5)	0.95 (0.62–1.47)	14 (12.4)	226 (10.0)	1.08 (0.59–1.97)	13 (11.5)	261 (11.5)	0.94 (0.51–1.72)
[0–24] months	57 (50.4)	974 (43.1)	1.38 (0.94–2.03)	30 (26.5)	382 (16.9)	**1.87 (1.21–2.89)**	26 (23.0)	441 (19.5)	1.28 (0.81–2.03)
**PMR (cases** = **232, controls** = **4640)**									
[0–12] months	70 (30.2)	1281 (27.6)	1.14 (0.85–1.54)	25 (10.8)	477 (10.3)	1.10 (0.70–1.71)	26 (11.2)	553 (11.9)	0.92 (0.60–1.41)
[12–24] months	68 (29.3)	1326 (28.6)	1.01 (0.75–1.36)	20 (8.6)	453 (9.8)	0.86 (0.53–1.41)	28 (12.1)	554 (11.9)	1.03 (0.60–1.55)
[0–24] months	114 (49.1)	2053 (44.2)	1.23 (0.94–1.61)	38 (16.4)	776 (16.7)	0.99 (0.69–1.41)	49 (21.1)	963 (20.7)	1.02 (0.74–1.42)

*Note*: Significant values are indicated in bold.

Abbreviations: 95% CI, 95% confidence interval; aOR, adjusted odds ratio.

^a^ Analyses adjusted for educational level, socio‐professional category, body mass index, smoking status, Type 2 diabetes, history of cancer.

No association was found for PMR, but in analyses restricted to GCA, we found an even stronger positive association with beta‐lactam reimbursements in the [0–24] month period (aOR [95% CI] 1.38 [0.94–2.03]) and with quinolone reimbursements in the [0–12] (aOR [95% CI] 2.07 [1.23–3.49]) and [0–24] (aOR [95% CI] 1.87 [1.21–2.89]) month periods.

### Sensitivity analysis

The date of initiation of the first >3‐month course of GC within a 1‐year period around index date was available for 406 (94.9%) cases, who were compared to their 8120 matched controls.

When this date was considered the index date for cases, results were comparable to the primary analysis (Appendix S2). Antibiotic use was associated with the risk of GCA/PMR in the [0–12] month period (aOR [95% CI] 1.24 [1.00–1.52]). Considering the [0–6] month and [0–24] month periods, the association was nearly significant (aOR [95% CI] 1.19 [0.96–1.48] and 1.20 [0.97–1.48], respectively). Similarly, this association was only found with GCA cases on those same periods, but not with PMR cases.

## Discussion

In this nested case–control study within a large prospective cohort of French women, we found a positive association between any antibiotic reimbursements and the occurrence of GCA/PMR. This association was only found among GCA cases, but not when PMR cases were considered separately. In addition, this risk was only found when the closest period prior to index date was included, suggesting that infections could be a trigger of the diseases. We also analyzed different antibiotic classes separately, revealing a strong association with the use of quinolones, and we considered the date of the first GC reimbursement as the index date to reduce the reverse causation bias.

The association between infections and the occurrence of GCA and/or PMR has been previously studied with different exposure definitions (mainly by coding data) and with different assessed periods [18, 19, 21, 32]. Our results are consistent with those from a nested case–control study from Rhee et al. [19], who showed that the association between infection and GCA onset was stronger when infection occurred in the year prior to index date. Similarly, Stamatis et al. [18], through a case–control study, found a significant association between prior infections and GCA, which was stronger when infections close to index date were taken into account. Only one other study by Brault et al. assessed the association between prior infections with both GCA and PMR [21], through two mutually exclusive time periods. Similarly, the risk was higher when infections occurring ≤1 year prior to index date were considered, especially, and like in our study, for GCA. This association was no longer found with PMR for infections occurring >1 year prior to index date. In our study, we considered multiple periods of exposure to precise the timing of the association and found a higher risk for infections that were close to the index date (i.e., within 0–6 months before the diagnosis).

The hypothesis that GCA/PMR would be triggered by an infectious agent has already been put forward. Particularly in GCA, the inflammatory reaction within the artery wall is suspected to be an inappropriate excessive reaction to antigenic stimulation [33], which may include infectious triggers. The increased risk we have shown with prior infections close to diagnosis might support this hypothesis. Nevertheless, a reverse‐causation bias and a surveillance bias cannot be excluded. Indeed, prior infections close to diagnosis could have been misclassified and be one of the first signs of GCA/PMR, such as fever or elevated C‐reactive proteins, leading to more microbiological tests and antibiotic prescriptions within cases compared to controls.

Interestingly, this association was observed for GCA but not for PMR, which may reflect differences in their underlying immunopathogenic mechanisms. In GCA, dendritic cell activation via Toll‐like receptors and subsequent Th1/Th17 polarization—processes influenced by microbial signals and potentially by dysbiosis—are central to disease development [8, 34]. In contrast, PMR is mainly characterized by systemic, IL‐6‐driven inflammation without vascular dendritic cell involvement, which may explain the absence of association with prior antibiotic exposure [35, 36].

On the other hand, GC treatment could induce immunosuppression and increase the risk of infection. To our knowledge, none of the previous studies analyzed the exact timing of GC therapy. In our work, we accounted for this risk in our sensitivity analysis, where the first prescription of GC was considered index date, and found similar results, reducing the risk of reverse causation bias.

In addition, despite a reduced statistical power (due to a lower number of GCA cases), we found that quinolone reimbursement was strongly and significantly associated with diagnosis of GCA, with, once again, an increased risk when the reimbursement was close to the index date. To our knowledge, this is the first study that separately analyzed the association with different classes of antibiotics. Altered microbiome composition has been implicated in inflammatory diseases such as inflammatory intestinal disease, rheumatoid arthritis [37], and all‐sized vessel vasculitis [38]. Moreover, the impact of quinolones on microbiome has already been highlighted [39]. In GCA, no study focused specifically on the intestinal microbiome but rather on the blood and large vessel microbiome. Blood samples from GCA patients compared with healthy donors showed differences, such as an increased abundance of *Rhodococcus* and an unidentified member of the Cytophagaceae family [40]. Differences were also observed comparing temporal arteries from GCA patients and non‐GCA controls, with varying abundances of *Proteobacteria, Bifidobacterium, Parasutterella*, and *Granulicatella* [41]. Although it is well established that beta‐lactam antibiotics exert a broad and substantial impact on the gut microbiome, particularly on anaerobic species, evidence also suggests that quinolones can significantly alter gut microbial composition, with some studies reporting a longer persistent dysbiosis and reduced microbial diversity, notably affecting specific Gram‐negative subpopulations and certain anaerobes. It is therefore plausible that different classes of antibiotics exert distinct effects on the gut microbiota, which may differentially influence the risk of immune‐mediated diseases such as GCA. We could therefore hypothesize that these selected bacterial changes—potentially driven by the impact of quinolones—may act as antigenic triggers in the pathogenesis of GCA, or that GCA could reflect an aberrant immunological response to a compromised microbiome.

We acknowledge some limitations to our study. First, because data from reimbursement claims database were only available from 2004 onward, we could not assess the risk between infections and GCA/PMR over longer periods of time. For the same reasons, we could not include cases diagnosed before 2006. However, only a minority (21.9%) of our cases were diagnosed prior to 2006. In addition, our cohort is made up entirely of women, making it impossible to generalize to men. Moreover, this is a specific population of women working in the French education system, which may have influenced certain findings and should be considered when interpreting the extent to which the results can be generalized to other populations. Nevertheless, as GCA/PMR mostly affects women (3:1), our study population was appropriate to study this association. Moreover, as previously mentioned, because GCA may present as a febrile illness, we cannot rule out a reverse‐causality bias, particularly given that the highest OR was observed for antibiotic treatment administered close to the onset of GCA. Finally, our exposure definition relied on antibiotic reimbursements only and not on clinically defined episodes. Thus, we cannot be sure they were bacterial infections, and it is highly probable that some were viral infections, because infections may have been treated without documentation and antibiotics might have been overused [42]. We could not consider infections that did not require antibiotics and infections that were completely treated at the hospital, as hospital‐delivered antibiotics were not registered in our reimbursement database. Therefore, our measure of exposure may be subject to misclassification. However, such misclassification is likely nondifferential with respect to case/control status, which would tend to bias results toward the null.

Nevertheless, this study has some strengths. First, it comes from a large prospective cohort of women, with very few missing data and extensive available data. Thus, compared with coding data, we could adjust our analyses on BMI, smoking status, educational level, and socio‐professional categories, which are often unavailable in population‐based registries. In addition, GCA cases were validated using a specific questionnaire, which has high specificity and precise data on the date of diagnosis, in contrast to other epidemiological studies based mainly on coding data. Moreover, we have analyzed the association between infections and further GCA/PMR over different time periods. We minimize the risk of reverse causation bias mediated by prior GC intakes by analyses using the first intake as index date. Finally, to our knowledge, this is the first study assessing the impact of antibiotic class on further GCA/PMR.

In conclusion, this nested case–control study demonstrated an increased likelihood of GCA/PMR following any infections. Risk was mainly for GCA cases and was higher when infections occurred close to diagnosis date, supporting the idea of an infectious antigenic trigger. Nevertheless, a reverse causation bias cannot be excluded. Moreover, despite a reduced statistical power, prior quinolone use was strongly and significantly associated with further diagnosis of GCA, which could raise the question of the implication of a disbalanced microbiome in the pathogenesis of GCA.

## Author contributions

Lucas Pacoureau, Yann Nguyen, and Raphaèle Seror designed the study. Lucas Pacoureau and Yann Nguyen performed the data management and statistical analyses. Lucas Pacoureau wrote the first draft of the manuscript. All authors reviewed the manuscript and approved the final version.

## Conflict of interest statement

L.P., F.B., A.F., A.E., and Y.N. declare no competing interests. R.S. received consulting fees from GSK, Bristol Myers Squibb, Boehringer, and Janssen; honoraria from GSK, Bristol Myers Squib, Boehringer, Amgen, Pfizer, and Roche; and travel fees from Amgen and GSK, outside of the topic of this study.

## Funding information

This work has been supported as part of France 2030 program “ANR‐11‐IDEX‐0003”, from the OI HEALTHI of the Université Paris‐Saclay for master's degree grant (L.P.), and by the Fondation pour la Recherche Médicale (SPF202209015782) for post‐doctoral grant (Y.N.). This work was realized with data of the E3N cohort (Inserm) and supported by the Mutuelle Générale de l'Education Nationale (MGEN), Gustave Roussy Institute, and French League against Cancer for the constitution and maintenance of the cohort. This work has benefited from State aid managed by the National Research Agency (ANR) under the program “Investment in the future” bearing the reference ANR‐10‐COHO‐0006, as well as a subsidy from the Ministry of Higher Education, Research and Innovation for public service charges bearing the references N°2102918823, 2103236497, and 2103586016.

## Ethics statement

Approval was obtained from the French National Commission for Data Protection and Individual Freedom (327346‐V14) and the French Advisory Committee on Information Processing in Material Research in the Field of Health (13.794).

## Consent

All participants signed an informed consent form at study entry.

## Supporting information




**Supplementary Appendix 1**: Antibiotics ATC classification.
**Supplementary Appendix 2**: Sensitivity analysis: association between antibiotics consumption by period and incident cases of GCA/PMR, GCA or PMR alone, taking the date of first glucocorticoid reimbursement as index date.
