# Supplementary material for: Association between antibiotic use and the onset of giant cell arteritis and polymyalgia rheumatica: A nested case–control study from E3N‐European Prospective Investigation into Cancer and Nutrition
Source: J Intern Med. 2025 Sep 2;298(5):424–37. doi: 10.1111/joim.70000 (PMC12522520; doi:10.1111/joim.70000)
Supplement: Supplementary file 1 — Supplementary Appendix 1: Antibiotics ATC classification. Supplementary Appendix 2: Sensitivity analysis: association between antibiotics consumption by period and incident cases of GCA/PMR, GCA or PMR alone, taking the date of first glucocorticoid reimbursement as index date. [file JOIM-298-424-s001.docx]

SUPPLEMENTARY MATERIAL

**Supplementary appendix 1. Antibiotics ATC classification**

| **Antibiotics class** | **Antibiotic name** |
| --- | --- |
| Tetracyclines J01AA | J01AA02 Doxycycline  J01AA03 Chlortetracycline  J01AA04 Lymecycline  J01AA05 Metacycline  J01AA06 Oxytetracycline  J01AA07 Tetracycline  J01AA08 Minocycline  J01AA20 Combinations of tetracyclines  QJ01AA53 Chlortetracycline, combinations  J01AA56 Oxytetracycline, combinations |
| Penicillins with extended spectrum J01CA* | 01CA02 Pivampicillin  J01CA04 Amoxicillin  J01CA06 Bacampicillin  J01CA08 Pivmecillinam |
| Beta-lactamase-sensitive penicillins J0ICE* | J01CE01 Benzylpenicillin  J01CE02 Phenoxymethylpenicillin  J01CE08 Benzathine benzylpenicillin  J01CE10 Benzathine phenoxymethylpenicillin |
| Beta-lactamase-resistant penicillins JOICF* | J01CF02 Cloxacillin  J01CF04 Oxacillin  J01CF05 Flucloxacillin |
| Combinations of penicillins, including beta-lactamase inhibitors J01CR* | J01CR02 Amoxicillin and beta-lactamase inhibitor |
| First-generation cephalosporins J01DB* | J01DB01 Cefalexin  J01DB05 Cefadroxil  J01DB09 Cefradine |
| Second-generation cephalosporins J01DC* | J01DC01 Cefoxitin  J01DC02 Cefuroxime  J01DC04 Cefaclor  J01DC07 Cefotiam |
| Third generation cephalosporins J01DD* | J01DD01 Cefotaxime  J01DD04 Ceftriaxone  J01DD08 Cefixime  J01DD13 Cefpodoxime  J01DD51 Cefotaxime and beta-lactamase inhibitor  J01DD54 Ceftriaxone, combinations  J01DD63 Ceftriaxone and beta-lactamase inhibitor |
| Combinations of sulfonamides and trimethoprim J01E | J01EA Trimethoprim and derivatives  J01EA01 Trimethoprime  J01EC Intermediate-acting sulfonamides  J01EC01 Sulfamethoxazole  J01EC02 Sulfadiazine  J01EE Combinations of sulfonamides and trimethoprim, including derivatives  J01EE01 Sulfamethoxazole and trimethoprim J01EE02 Sulfadiazine and trimethoprim |
| Sulfonamides QJ01EQ | QJ01EQ10 Sulfadiazine  QJ01EQ11 Sulfamethoxazole |
| Combinations of sulfonamides and trimethoprim, including derivatives QJ01EW | QJ01EW10 Sulfadiazine and trimethoprim  QJ01EW11 Sulfamethoxazole and trimethoprim |
| Macrolides J01FA¶ | J01FA02 Spiramycin  J01FA03 Midecamycin  J01FA06 Roxithromycin J01FA07 Josamycin  J01FA09 Clarithromycin  J01FA10 Azithromycin  J01FA13 Dirithromycin |
| Lincosamides J01FF¶ | J01FF01 Clindamycine  J01FF02 Lincomycine  QJ01FF52 Lincomycine, combinations |
| Streptogramins J01FG¶ | J01FG01 Pristinamycin |
| Fluoroquinolones J01MA ¶ | J01MA01 Ofloxacin  J01MA02 Ciprofloxacin  J01MA03 Pefloxacin  J01MA04 Enoxacin  J01MA06 Norfloxacin  J01MA07 Lomefloxacin  J01MA12 Levofloxacin  J01MA14 Moxifloxacin  J01MA16 Gatifloxacin |
| Other quinolones J01MB | J01MB04 Pipemidic acid J01MB07 Flumequine |
| Combinations of antibacterials J01RA | J01RA01 Penicillins, combination with other antibacterials*  J01RA02 Sulfonamides, combination with other antibacterials  (excluding trimethoprim)  J01RA03 Cefuroxime and metronidazole  J01RA04 Spiramycin et metronidazole  J01RA05 Levofloxacin and ornidazole  J01RA09 Ofloxacin and ornidazole  J01RA10 Ciprofloxacin and metronidazole  J01RA11 Ciprofloxacin and tinidazole  J01RA12 Ciprofloxacin and ornidazole  J01RA13 Norfloxacin and tinidazole  QJ01RA90 Tetracycline, combination with other antibacterials  QJ01RA91 Macrolides, combination with other antibacterials  QJ01RA94 Lincosamides, combination with other antibacterials  QJ01RA96 Quinolones, combination with other antibacterials |
| Polymyxins J01XB | J01XB02 Polymyxin B |
| Steroid antibacterials J01XC | J01XC01 Fusidic acid |
| Imidazole derivatives J01XD | J01XD01 Metronidazole  J01XD02 Tinidazole  J01XD03 Ornidazole |
| Nitrofuran derivatives J01XE | J01XE01 Nitrofurantoin  J01XE51 Nitrofurantoin, combinations |
| Other antibacterials J01XX | J01XX01 Fosfomycin  J01XX06 Mandelic acid  J01XX08 Linezolid  J01XX10 Bacitracin  J01XX11 Tedizolid |
| *Included in the beta-lactam exposure, ¶ included in the macrolide exposure | |

**Supplementary appendix 2. Sensitivity analysis: association between antibiotics consumption by period and incident cases of GCA/PMR, GCA or PMR alone, taking the date of first glucocorticoid reimbursement as index date**

|  | **Overall** | | | | **GCA** | | | | **PMR** | | | |
| --- | --- | --- | --- | --- | --- | --- | --- | --- | --- | --- | --- | --- |
| **Time period prior to index date** | Cases  N=406 | Controls  N=8,120 | Univariate analysis  OR (95% CI) | Multivariate analysis*  OR (95% CI) | Cases  N=107 | Controls  N=2,140 | Univariate analysis  OR (95% CI) | Multivariate analysis*  OR (95% CI) | Cases  N=218 | Controls  N=4,360 | Univariate analysis  OR (95% CI) | Multivariate analysis*  OR (95% CI) |
| **By 6 months** | | | | | | | | | | | | |
| [0–6[ months | 124 (30.5) | 2,208 (27.2) | 1.20 (0.96 – 1.50) | 1.19 (0.96 – 1.48) | 45 (42.1) | 596 (27.9) | **1.89 (1.27 – 2.82)** | **1.94 (1.27 – 2.94)** | 63 (28.9) | 1,196 (27.4) | 1.08 (0.80 – 1.46) | 1.07 (0.78 – 1.47) |
| [6–12[ months | 111 (27.3) | 2,162 (26.6) | 1.02 (0.81 – 1.29) | 1.05 (0.84 – 1.31) | 35 (32.7) | 540 (25.2) | 1.44 (0.95 – 2.19) | 1.34 (0.86 – 2.09) | 62 (28.4) | 1,193 (27.4) | 1.06 (0.78 – 1.43) | 1.06 (0.77 – 1.46) |
| [12–18[ months | 113 (27.8) | 2,192 (27.0) | 1.05 (0.83 – 1.32) | 1.06 (0.85 – 1.32) | 27 (25.2) | 559 (26.1) | 0.96 (0.61 – 1.49) | 0.84 (0.53 – 1.35) | 67 (30.7) | 1,226 (28.1) | 1.14 (0.84 – 1.53) | 1.16 (0.85 – 1.58) |
| [18–24] months | 104 (26.6) | 2,204 (27.1) | 0.89 (0.70 – 1.13) | 0.93 (0.74 – 1.17) | 29 (27.1) | 573 (26.8) | 1.02 (0.66 – 1.57) | 0.92 (0.58 – 1.46) | 56 (25.7) | 1,219 (28.0) | 0.89 (0.65 – 1.22) | 0.84 (0.61 – 1.17) |
| **By 12 months** | | | | | | | | | | | | |
| [0–12[ months | 190 (46.8) | 3,421 (42.1) | 1.21 (0.99 – 1.48) | **1.24 (1.00 – 1.52)** | 58 (54.2) | 899 (42.0) | **1.65 (1.11 – 2.44)** | **1.76 (1.18 – 2.64)** | 103 (47.2) | 1,870 (42.9) | 1.20 (0.91 – 1.58) | 1.23 (0.92 – 1.63) |
| [12–24] months | 171 (42.1) | 3,450 (42.5) | 0.99 (0.80 – 1.21) | 0.95 (0.77 – 1.17) | 45 (42.1) | 890 (41.6) | 1.02 (0.69 – 1.51) | 0.92 (0.62 – 1.39) | 95 (43.6) | 1,910 (43.8) | 0.99 (0.75 – 1.30) | 0.95 (0.72 – 1.26) |
| **By 24 months** | | | | | | | | | | | | |
| [0–24] months | 264 (65.0) | 4,961 (61.1) | 1.19 (0.96 – 1.46) | **1.20 (0.97 – 1.48)** | 74 (69.2) | 1,308 (61.1) | 1.43 (0.94 – 2.19) | 1.48 (0.97 – 2.27) | 143 (65.6) | 2,706 (62.1) | 1.17 (0.88 – 1.56) | 1.18 (0.88 – 1.57) |
| **Analyses adjusted for educational level, socio-professional category, body mass index, smoking status, type 2 diabetes, history of cancer*  *Abbreviations: OR=odds ratio; 95% CI=95% confidence interval; GCA=giant cell arteritis; PMR=polymyalgia rheumatica* | | | | | | | | | | | | |
